# Wesley J. Thompson (1947–2019)

**DOI:** 10.3389/fnmol.2020.00091

**Published:** 2020-06-12

**Authors:** Young il Lee, Mendell Rimer

**Affiliations:** ^1^Department of Biology, Texas A&M University, College Station, TX, United States; ^2^Department of Neuroscience and Experimental Therapeutics, Texas A&M University Health Science Center, Bryan, TX, United States

**Keywords:** Wesley J. Thompson, neuromuscular junction (NMJ), synapse elimination, schwann cell, activity-dependent synaptic plasticity, nestin, neuregulin1, muscle fiber development

Wesley Jay (Wes) Thompson, known for his seminal contributions to the studies of neuromuscular synapses and glial cells, passed away on March 26, 2019, at the age of 71. Surviving family, friends, trainees, colleagues, and others who hold Wes in fond memory gathered at the campus of Texas A&M University in College Station, TX, for a memorial service in his honor some weeks later. All of the speakers recounted with fondness their interactions with Wes that exemplified the impact his approaches to life and work had on the lives of many others. Most who knew Wes would agree that he never liked to talk about himself much except in performing self-deprecating humor and voicing his displeasure with the current political climate. Wes abhorred any public recognition of his accomplishments (or good deeds) and only begrudgingly tolerated those accompanied by research funds. We are certain Wes would have protested the production of this article that pays him tribute. There are aspects of his life, however, that were readily apparent to those who have spent any meaningful amount of time with Wes—his generosity, drive and integrity as a scientist, affinity for history and simpler things in life such as his vegetable garden and backyard barbeque with friends that brought him joy, and love for his daughter. Jeff Lichtman, a fellow towering contemporary figure in the study of neuromuscular synapses, once called Wes “a gem of a human being,” a much-deserved compliment that sweetly sums up the warm character of the man we all knew.

Wes was born on December 10, 1947, in Alice, TX, to Jay and Harriett Thompson from whom Wes inherited many of his endearing traits: rough-around-the-edges Texan charm, a desire/willingness to fix any mechanical instruments (often with mixed results) from his inventor/oilman father, and a deep belief in the value of education from his mother, a school teacher. The family relocated to Crawford, TX, where Wes spent a large part of his childhood. In 1966, Wes graduated from Crawford High School, and in 1970, earned a Bachelor of Science degree with High Honors from North Texas State University (since renamed the University of North Texas), a short 2-h drive away. Perhaps as a preview of what lay ahead in his long and respected scientific career punctuated with honors, a survey of his high school and college yearbooks (Figure 1) provides hints that fellow students had already recognized Wes's academic accomplishments and potential. As his career progressed, clearly, Wes did not fall victim to enemies of promise. In addition to receiving several postdoctoral fellowships, he was named a Searle Scholar in 1981 (Searle Scholars Program, Chicago Community Trust) and received a National Institutes of Health (NIH) Research Career Development Award in 1984 and a Javits Neuroscience Investigator award in 2001. He maintained a research program that was continuously funded by the NIH until his death. As a testament to his long-standing dedication to educate undergraduate students, Wes was also recognized with a University of Texas at Austin Teaching Excellence Award in 2000.

**Figure 1:**
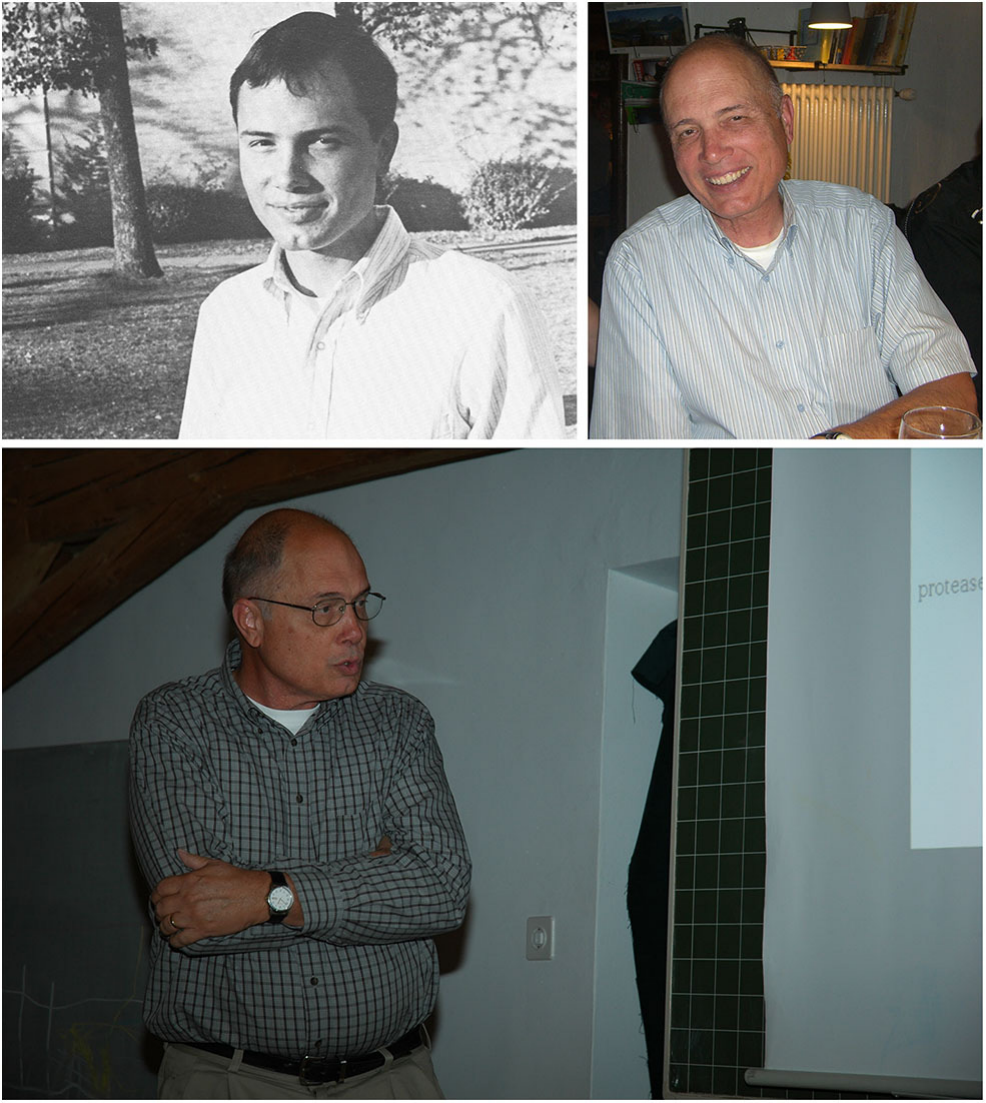
(**Top left**) Wes at North Texas State University (1966–1970). As a senior, when he was recognized for outstanding work in his major field of study—Biology. Source: The Yucca, Yearbook of North Texas State University, 1970, Fort Worth, TX; University of North Texas Libraries, UNT Digital Library (https://digital.library.unt.edu), crediting UNT Libraries Special Collections. (**Bottom**) Wes, in his customary presentation stance, at the 2010 biennial “Molecular and Cell Biology of the Neuromuscular System” meeting, organized by Hans Brenner, Stephan Kröger, and Markus Rüegg, Guarda, Switzerland. Photograph kindly provided by Dr. Shuo Lin, University of Basel, Switzerland. (**Top right**) Wes at a dinner during the 2012 Guarda meeting. From an original photograph kindly provided by Dr. Noreen Reist, Colorado State University, USA.

## The Journey West

After submitting his thesis for a Master of Arts degree at North Texas State University in December 1971, Wes headed west to pursue graduate studies at the University of California, Berkeley. Early 70s Berkeley, CA, was probably as foreign a place as could be imagined for a young man from central Texas. At Berkeley, Wes joined the lab of Gunther Stent, a disciple of the “phage group” led by Caltech's Max Delbrück, whose research on the genetic structure and replication of bacterial viruses (bacteriophages) helped launch the modern field of Molecular Biology. By the time Wes joined his lab, Gunther Stent had famously declared Molecular Biology near dead as a field and had moved on to Neurobiology using the medicinal leech, *Hirudo medicinalis*, as a model organism for investigating the neurobiological basis of behavior. With its relatively simple and accessible nervous system, the leech offered an excellent experimental model to characterize and understand the neuronal circuits that underlie rhythmic movements such as swimming or crawling (Kristan et al., 2005). While the rest of the Stent group was focused on finding the neuronal circuitry that produced swimming in the leech, for his doctoral thesis, Wes tackled the regulation of heartbeat. The leech heartbeat consists of neuronally driven, pulsing contractions of muscular lateral, longitudinal vessels, known as the heart tubes, which drive blood flow through a closed circulatory system (Kristan et al., 2005). Wes carried out pioneering work that first described the innervation circuit of the heart tubes by excitatory motor neurons (HE cells) (Thompson and Stent, 1976a). Most importantly, Wes discovered the ensemble of inhibitory heart interneurons (HN cells) that constitute the oscillator circuit driving the bursting firing pattern of the HE cells, which generates the heartbeat (Thompson and Stent, 1976b,c). Wes characterized the mechanism of the oscillation as the reciprocal inhibition between HN cells, how the oscillatory pattern was imposed on the HE cells, and how the hearts on the two sides of the leech were coordinated. This work provided the basis for later, elegant studies by Ron Calabrese et al. that have advanced in great detail the understanding of the neuronal circuitry controlling this behavior (Calabrese et al., 2016). The leech heartbeat system, the heartbeat circuitry in the lobster, and the control of the stomatogastric systems of lobsters and crabs are among the very few completely characterized oscillator circuits in all of neuroscience. Bill Kristan, then a postdoctoral fellow in the Stent lab, who became an eminent figure in leech biology and the neurobiology of behavior, has observed that Wes's thesis work “required a huge amount of very careful experiments along with great insight at many stages of the project. I could tell when he was being most productive because he would take minimal time to satisfy creature needs like eating and drinking. His lunches and dinners during these times consisted almost entirely of candy bars and cokes!” Stent and co-disciples, affectionately known as Stent's “neurobiological minions” (Weisblat and Thompson, 2008), influenced Wes's intellectual and practical approach to science for the rest of his career. Kristan, in particular, played an important mentoring role during Wes's time in the Stent lab, and they remained close friends and colleagues throughout Wes's life.

## To Oslo, Norway

One of us once asked Wes what compelled him to move all the way to Oslo, Norway, to join the laboratory of Jan Jansen for a postdoc. His sheepish answer, delivered with his characteristic half-smile, was that Stent had already decided for him and he “didn't have a say in the matter.” Wes's work with Jansen in the late 70s was foundational to the study of activity-dependent plasticity, and laid the foundation for Wes's work for the rest of his career. When Wes arrived at the University of Oslo, experiments had just been completed that described a dramatic and puzzling change from innervation of individual rat muscle fibers by several different motor neurons at birth to innervation by a single motor neuron some 2 weeks later (Brown et al., 1976). The process was named “synapse elimination.” At the Oslo institute, Wes overlapped with Terje Lømo, a pioneer in studying activity-dependent plasticity in the nervous system. Influenced by ideas and works by Stent and Lømo, Wes was well aware of the important role neural activity played in the developing nervous system, particularly in the maturation of muscle fibers and their synaptic innervation. He thus spent his time in the Jansen lab, and much of his independent career, studying how activity shaped developmental synapse elimination using rodent neuromuscular junctions (NMJs) as a model system. Wes, Damien Kuffler, and Jan Jansen were the first to demonstrate in a heroic series of *in vivo* experiments that blocking action potential conduction along nerves delayed or prevented synapse elimination in postnatal rats (Thompson et al., 1979). Perhaps the most important and impactful insight Wes and his work in Jansen's lab provided was that synapse elimination was a reflection of an active, competitive process among motor neurons innervating the same NMJ, rather than a passive, random process. Wes demonstrated this by surgically reducing the number of motor axons innervating a muscle shortly after birth, which led to a dramatic increase in the number of muscle fibers innervated by each of the spared motor neurons (Thompson and Jansen, 1977). The concept that developmental synapse elimination was competitive has been foundational to neuroscience and was further fleshed out in later studies by Lichtman and colleagues among others (Walsh and Lichtman, 2003; Schafer et al., 2012).

By reducing the number of motor neurons innervating skeletal muscles to understand the drivers of synapse elimination, Wes had also become interested in the process of sprouting, how the spared motor neurons expand their innervation of muscle fibers transiently denervated subsequent to injury. Wes found there was a limit to how much individual remaining motor neurons could expand their innervation. In the jargon of the field, he discovered that there was an upper limit to motor unit size which was about five times the typical number of muscle fibers innervated by a single motor neuron (Thompson and Jansen, 1977). Wes's findings also set a lower limit on how many motor neurons could be lost before muscle function was compromised. This work continues to have important implications for understanding neuromuscular diseases and injury, and the impact of these on muscle function, themes to which Wes and his lab would return later in his career.

## Return to Texas

Despite the lack of a prominent Texas drawl, Wes remained a Texan through and through. He longed to return to his home state as an independent scientist. Returning to the United States, Wes did a second postdoc in the laboratory of Dale Purves at Washington University in St. Louis. While in the Purves lab, Wes studied the reestablishment of synaptic connections after nerve injury in sympathetic ganglia as a model system (Purves and Thompson, 1979; Purves et al., 1981).

While in St. Louis, Wes would befriend Jeff Lichtman, who was a graduate student in the Purves lab at the time and would himself become a leader in the study of the role of activity as a modulator of synapse elimination. The two would spend the next three decades working on this topic, Wes at the University of Texas, later at Texas A&M University, and Jeff at Washington University and later at Harvard. These pioneering scientists produced many of the landmark studies that extend our understanding of the role of activity in shaping neural circuitry during development and plasticity in the reestablishment of innervation following nerve or muscle injury.

Wes's statement of his research interests, provided by the Searle Scholar Program, which recognized him as an exceptional young faculty, exemplifies Wes's style of communication—clear, to the point, and exceedingly modest. It reads: “Formation and Maintenance of Synaptic Connections: I am interested in the formation and maintenance of synaptic connections in the developing nervous system. In particular, I am investigating the remodeling [sic] of neuromuscular synapses which occurs in mammalian muscles during late fetal and early postnatal stages. I wish to understand how the different kinds of motor neurons and muscle fibers achieve their final differentiation and how the motor neurons come to selectively innervate the appropriate muscle fibers. In the course of this work, my lab has generated antibodies which recognize a novel component of the NMJ. An additional objective is to determine the identity of this component and its role in the differentiation of this synapse.”

## The University of Texas Years

After about a year in the Purves lab, in 1979, Wes established his own research lab at the University of Texas in Austin, where his work continued to advance our understanding of how activity influences developmental synapse elimination using NMJs as a model system. By this time, it was well-recognized, from Wes's work and that of others, that the absolute levels of activity profoundly impacted the time course of neuromuscular synapse elimination. In his seminal 1983 *Nature* paper, Wes elegantly demonstrated that the activity patterns with which muscle fibers were stimulated shaped the time course of synapse elimination in developing muscles (Thompson, 1983). This was a key observation that suggested that pre- and postsynaptic activities were crucial drivers of competition. Additionally, his report lent support to the hypothesis that competitive synapse elimination occurred *via* a Hebbian mechanism: coordinate pre- and postsynaptic activity strengthened synapses, while dis-coordinate activity weakened synapses, perhaps, thus driving their loss. This hypothesis was tested in various ways over the years that followed, by Wes, his colleagues, and other labs, ultimately leading to the demonstration that the relative timing of action potentials impacts profoundly synaptic strength and synapse loss at neuromuscular (Personius and Balice-Gordon, 2001; Buffelli et al., 2002) and other synapses (e.g., Lorenzetto et al., 2009; Zhang et al., 2011).

Prior to Wes's work, programmed motor neuron cell death and muscle fiber addition during development had been proposed to drive neuromuscular synapse elimination (Harris, 1981; Nurcombe et al., 1981; Bennett et al., 1983). It had been argued that because motor neuron death preceded the removal of distal terminal axonal branches, downstream loss of their synapse with muscle fibers would inevitably occur. Similarly, because muscle fibers increase in number during early postnatal life, the hypothesis was posited that the postnatal emergence of new fibers would result in the shifting of synapses from multiple innervated fibers to new, as yet uninnervated fibers. Such change in synapse distribution could be mischaracterized as synapse elimination. Wes observed alterations to neither the number of motor units (the functional readout of the number of innervating motor neuron) nor the number of muscle fibers within a target muscle during the postnatal period of synapse elimination (Balice-Gordon and Thompson, 1988b). He further showed that the tension generated by individual motor units decreased during this period, consistent with previous work (Brown et al., 1976). Wes's findings showed that each motor neuron reduced the number of muscle fibers it innervated as a consequence of synapse elimination, ruling out a change in motor neuron or muscle fiber number as factors in this process.

Despite the heterogeneity of muscle fiber types (e.g., defined by myosin heavy chain expression and/or contractile speed), each mature motor unit contains only a single muscle fiber type innervated by a motor neuron, whose firing pattern is functionally matched to the muscle fiber's contractile properties. Wes and others had demonstrated that motor unit fiber type homogeneity is present prior to the completion of synapse elimination (Thompson et al., 1984; Gordon and Van Essen, 1985; Balice-Gordon and Thompson, 1988b). To this day, it remains unclear how a homogeneous group of muscle fibers comes to reside within each mature motor unit. Despite variation in levels and pattern of activity, the contractile property of a given muscle—and even more remarkably, the distribution of fiber types within a muscle—shows limited variability among individuals of a species. Wes's demonstrations of the profound influence neuromuscular activity pattern has on muscle fiber contractile properties, in addition to the timing of neuromuscular synapse elimination (Thompson, 1983), raised an obvious question: What is the extent of muscle fiber autonomy in fiber type differentiation? To address this question, Wes needed antibodies that would differentiate muscle fiber types, which at the time were not available. He went about generating monoclonal antibodies—a substantial undertaking in the 1980s, and he even attended a Cold Spring Harbor Laboratory course on how to do so. While at Cold Spring Harbor Laboratory, Wes met Laura Silberstein, then a postdoc with Helen Blau, who would share with him the necessary antibody reagents to facilitate his experiments. Because innervation of adult muscles by foreign nerves (or direct stimulations that mimic such foreign innervation) resulted in dramatic changes in muscle fiber types (Buller et al., 1960; Lømo et al., 1974; Thompson, 1983), it had been assumed that developmental muscle fiber type differentiation was also innervation- and activity-dependent. Instead, Wes showed that muscle fiber type differentiation, and the pattern of fiber type distribution within developing muscles, occurred normally even in the absence of innervation (Condon et al., 1990). He also showed that while some muscles eventually degenerated if permanently denervated during development, in agreement with previous studies (e.g., Harris, 1981), secondary myogenesis occurred with normal timing in muscles that persisted. These findings, thus, illustrated a surprisingly significant degree of autonomy in the generation and differentiation of muscle fibers. In addition, as muscle fiber types can differentiate independently of the nervous system, motor axons are paired with fiber types of appropriate contractile properties within predestined “compartments” of developing muscles (Balice-Gordon and Thompson, 1988a).

A fortuitous by-product of Wes's efforts to generate muscle fiber type-specific monoclonal antibodies was the generation of clones that would take his career on a picturesque, and highly productive, detour: Wes himself, on a number of occasions, commented that although he was trained as an electrophysiologist, he had become more of a morphologist. As it turned out, some of the antibodies Wes generated recognized the intermediate filament nestin, a protein localized postsynaptically at NMJs (Astrow et al., 1992). Upon denervation induced by nerve injury, however, nestin expression is suppressed in postsynaptic muscle fibers. Instead, its expression is turned on in the reactive Schwann cells (SCs) that form the bands of Büngner within the nerve segment distal to the injury site as well as in the SCs that localize to junctions, called terminal SCs (Astrow et al., 1994; Kang et al., 2007). These SCs exhibited elaborate process extensions, called sprouts (Reynolds and Woolf, 1992), similar in pattern to the axonal sprouts extended by regrowing motor axons. In a series of elegant papers in the mid-1990s, Wes and his colleagues discovered novel aspects of cell–cell interactions among motor axons and SCs that were essential for the establishment and maintenance of muscle innervation as well as reinnervation after injury. Wes demonstrated that terminal SCs and their processes both stimulated and guided regenerating motor axons back to denervated postsynaptic sites on muscle fibers in adult rodents (Son and Thompson, 1995a,b). Wes further showed that reinnervation of neonatal muscles is poor because of the dependence of regenerating motor axons on terminal SC processes: he found that denervation of neonatal muscles rapidly led to apoptotic death of terminal SCs and that denervation-induced SC apoptosis was prevented by injection of recombinant soluble neuregulin 1 (Trachtenberg and Thompson, 1996). Thus, this work demonstrated that neonatal SCs require neuregulin 1-dependent trophic support from motor axons, unlike the SCs in adult animals. The essential role of neuregulin 1 from motor axons in SC development *in vivo* suggested by this study was later confirmed and extended by mouse genetic experiments (Woldeyesus et al., 1999; Wolpowitz et al., 2000; Yang et al., 2001).

Desiring a more detailed understanding of the terminal SC sprouting response, Wes undertook the generation of transgenic mouse lines in which SCs expressed green fluorescent protein (GFP) (Zuo et al., 2004). Bred to another transgenic mouse, whose motor axons were labeled with a spectrally distinct (cyan) fluorescent protein (Feng et al., 2000), mice with fluorescent SCs allowed repeated vital imaging of motor axons and terminal SCs at NMJs in normal muscles, during denervation and subsequent reinnervation. This work led to the demonstration that SC sprouts actually preceded and led the outgrowth of motor axons during reinnervation. Wes further showed that the coverage of denervated synaptic sites by remaining terminal SCs significantly influences which sites are reinnervated (Kang et al., 2003, 2019). The creation of mouse lines with fluorescent SCs also led to other unanticipated, but nonetheless exciting and impactful observations. The transgene used to fluorescently label SCs (*S100-eGFP*) is also expressed in other cell types, including in central nervous system astrocytes and microglia. It thus became possible to isolate nearly-pure populations of astrocytes from the brains of these transgenic mice for transcriptomic analysis, a feat first accomplished by the late Ben Barres (Cahoy et al., 2008). The astrocyte expression database made possible by the “off-label” use of Wes's fluorescent glial mice has become an invaluable resource for countless neuroscientists all over the world. His trainees collectively delight in this inadvertent legacy of a neuroscientist who spent his career studying the peripheral nervous system and was determined to steer clear of the brain when conducting his work.

Wes's interest in NMJs extended to a quest to understand the mechanisms that underlie its morphological changes in aging and with diseases. The morphology of mature mammalian neuromuscular synapses is largely stable following the period of synapse elimination, with growth occurring by intercalary expansion as the muscle fibers grow. Several groups had observed that NMJs undergo conspicuous morphological changes as rodents age, becoming “fragmented” or “moth-eaten” in appearance, distinct from NMJs of young adult rodents (Balice-Gordon, 1997; Valdez et al., 2010; Li et al., 2011). It was believed that such dramatic change arose from incremental accumulation of small losses and additions of synaptic contacts at individual synapses, as mechanisms that ensure the stability of NMJs gradually waned with advancing age. However, following repeated *in vivo* imaging of NMJs from aged mice (Li et al., 2011), Wes found that junctional morphology is stable even in advanced age, with a large majority of junctions showing no changes to their morphology. He further found that a small fraction of junctions abruptly undergoes stochastic, wholesale morphologic changes, with the fraction of junctions that appear “fragmented” accumulating with age. He made the surprising observation that age-related morphologic changes in NMJs are instigated by the injury and subsequent regeneration of the innervated segment of muscle fibers. This was further corroborated by Wes's work that showed that similar rapid synaptic morphological changes occur in muscles from rodent models of Duchenne muscular dystrophy, as well as after deliberate muscle injury (Lyons and Slater, 1991; Li and Thompson, 2011; Haddix et al., 2018). Wes and his colleagues also studied mouse models of a severe form of the hereditary motor neuron disease spinal muscular atrophy (SMA). SMA results from low levels of the ubiquitously expressed protein survival of motor neuron (SMN). His group was among the first to demonstrate that, at least in these mice, SMA was not exclusively a motor neuron-autonomous disease, as specific muscles in SMA mice showed profound defects in neuromuscular development, even in the absence of any presynaptic deficits (Lee et al., 2011). With the recent approval of interventions that raise SMN levels in patient's motor neurons with ensuing remarkable clinical gains (Sumner and Crawford, 2018), Wes's work underscores the importance of continuing to study muscle function over time, to understand the contribution of postsynaptic muscle fibers to disease pathophysiology.

## The Texas A&M Years

In 2013, Jack McMahan, then head of the Biology Department at Texas A&M University, managed to convince Wes to move his lab “down the street” to College Station, TX, and join his department. And so, Wes became a Texas “Aggie” after more than 30 years as a fervent supporter of his beloved Texas “Longhorns.” For those in the know about Texas, Texans, and their traditions, this was a significant switch of allegiances for a born-and-bred Texan.

Wes's contributions while at A&M were as impactful as those earlier in his career. He used *in vivo* imaging of transiently denervated endplates to demonstrate that the degree to which reinnervation recapitulates the original synaptic morphology is inversely correlated with the duration of denervation. Wes noted that terminal SCs gradually retract their processes from endplate regions with prolonged denervation (Kang et al., 2014). The topology of the remaining terminal SCs and their processes was found to determine the branching pattern of returning motor axon terminals and the redistribution of postsynaptic acetylcholine receptors, thus providing a mechanistic explanation for the junction remodeling observed following nerve injury. Neuregulin 1, in addition to its role as a nerve-derived trophic factor for neonatal SCs, can induce responses in these cells *in vivo* that mimic responses to denervation and/or modify the morphology of NMJs (Trachtenberg and Thompson, 1997; Hayworth et al., 2006; Lee et al., 2016). Based on these findings, Wes believed it was a distinct possibility that neuregulin 1 signaling and SCs play important roles in neuromuscular synapse elimination. Indeed, genetic modulation of motor neuron-derived membrane-bound neuregulin 1 expression, which normally peaks during the first two postnatal weeks, shifts the time course of synapse elimination (Lee et al., 2016). An ultrastructural examination of early postnatal NMJs revealed two key features of terminal SCs that had previously gone unnoticed and unappreciated: the intercalation of their processes into the synaptic cleft and the phagocytic engulfment of motor axon terminals in contact with developing muscle fibers by these cells (Smith et al., 2013). These neuregulin 1-driven terminal SC responses are not observed at normal junctions beyond the period of synapse elimination (Lee et al., 2017). Collectively, Wes's work suggests a model in which terminal SCs randomly remove presynaptic motor nerve terminals, leading to the rapid reoccupation of the transiently abandoned postsynaptic receptor site by a nearby, competing motor nerve terminal. This hypothesis provides additional cellular and mechanistic context for the activity-dependence of synapse elimination. It further demonstrates that peripheral glia are active mediators of neuromuscular synapse elimination, as is the case with astrocytes and microglia during activity-dependent synapse elimination in the central nervous system (Neniskyte and Gross, 2017; Wilton et al., 2019).

## Embracing the “The Peanut Gallery”

Wes was passionate about science and committed to understanding how the nervous system develops and functions. His contributions have had a lasting impact in the fields of developmental and cellular neuroscience, providing fundamentally new insights into neuromuscular synapses in development and disease and revealing surprising facets of SC biology. Because of his efforts, we now have a better understanding of cellular and physiological mechanisms that promote developmental synapse elimination, the autonomy of muscle fiber development, the molecular nature of SC–motor neuron trophic interdependence, and the SC behaviors that promote efficacious reinnervation of target muscle fibers and any accompanying morphological changes to the synapse. Wes was creative: he never limited his approach to a particular experimental model or technique, instead inventing and/or adopting tools and techniques along the way that were essential to asking the right question. Success did not alter Wes's humility or generosity, two values which were core to his personality and for which he was much loved by all who knew him. Despite his many accomplishments and accolades, Wes always felt that he was privileged to be making a living as a research scientist. Perhaps because of the ever-increasing difficulty with which one might secure research funding, Wes would occasionally say, while worrying about grant proposals, that he could “go be a farmer.” Considering his affinity for growing things (in particular his love for and skill growing plumeria in his Texas garden), there is more than a grain of truth in those words. Yet, his passion for neuroscience—with his insightful, ask-the-right-question approach—was infectious. To the end of his life, Wes shared his love for a good research question—and the answer—with all who had the privilege of knowing him. Despite his penchant for playfully dismissing the lighthearted criticisms levied by his trainees and friends as noise from “the peanut gallery,” Wes encouraged others to speak with candor—especially about science. Equally generous with his time and advice as he was passionate about science, Wes remained a lifelong mentor, advocate, and friend to his many students and postdocs. Wes tirelessly encouraged and nurtured the careers of his trainees, as well as those of junior faculty members he had mentored at his own and other institutions. Many of his trainees and mentees have gone on to productive careers as scientists, and some became attorneys, educators, and physicians. Wes also selflessly and tirelessly served the larger neuroscience community for many years as a reviewer on NIH study sections and as an instructor for the renowned summer Neurobiology course at the Marine Biological Laboratory in Woods Hall, MA. All of us are deeply indebted to Wes for his mentorship, advice about science and life, encouragement, generosity, and most of all, for his friendship. We mourned his passing and remain grateful for the gifts Wes gave us and the foundation for future research that his life's work has provided to us and to the field.

We end this piece with a touching note of condolences from Bill Kristan, “Wes was a wonderful scientist—smart, creative, great experimentalist—and an even better person. He was gentle and humble, always concerned about others more than himself. He was a thoroughly dependable and enjoyable friend. The world has too few like him; we will miss him greatly.”

## Author Contributions

All authors listed have made a substantial, direct and intellectual contribution to the work, and approved it for publication.

## Conflict of Interest

The authors declare that the research was conducted in the absence of any commercial or financial relationships that could be construed as a potential conflict of interest.
